# Comparison of the usefulness of endoscopic papillary large-balloon dilation with endoscopic sphincterotomy for large and multiple common bile duct stones

**DOI:** 10.1186/s12876-015-0290-6

**Published:** 2015-05-16

**Authors:** Kouhei Tsuchida, Mari Iwasaki, Misako Tsubouchi, Tsunehiro Suzuki, Chieko Tsuchida, Naoto Yoshitake, Takako Sasai, Hideyuki Hiraishi

**Affiliations:** Department of Gastroenterology, Dokkyo Medical University, 880 Kitakobayashi, Mibu, Shimotsuga, Tochigi 321-0293 Japan

**Keywords:** Endoscopic papillary large-balloon dilation, Common bile duct stone, Endoscopic retrograde cholangiopancreatography

## Abstract

**Background:**

Endoscopic sphincterotomy (EST) is currently recognized as the primary endoscopic treatment for common bile duct stones. However, it is difficult to remove multiple (≥3) or large (≥15 mm) common bile duct stones with EST alone. Recently, EST plus endoscopic papillary large-balloon dilation (EPLBD) was reported to be an effective treatment for such bile duct stones. We compared the results of EST and EST + EPLBD for multiple (≥3) or large (≥15 mm) stones that were difficult to treat using EST alone. We also compared the complication rates between the techniques.

**Methods:**

Seventy patients with large (largest diameter, ≥15 mm) or ≥ 3 common bile duct stones treated in our department between April 2010 and March 2013 underwent EST + EPLBD (*n* = 34) or EST alone (*n* = 36). We compared final successful stone removal rates, rates of successful stone removal in the first session, procedure times, status of concurrent mechanical lithotripsy (ML), and complications between the EST + EPLBD and EST groups.

**Results:**

The rates of final successful stone removal were similar between the two groups (EST + EPLBD: 100 % vs. EST: 89 %; *p* = 0.115). The rate of successful stone removal in the first session was significantly higher in the EST + EPLBD group (EST + EPLBD: 88 % vs. EST: 56 %; *p* = 0.03). Moreover, the procedure time was significantly shorter (EST + EPLBD: 42 min vs. EST: 67 min; *p* = 0.011) and the rate of ML use was significantly lower in the EST + EPLBD group (EST + EPLBD: 50 % vs. EST: 94 %; *p* < 0.001). Complications like pancreatitis and bleeding occurred in three patients in the EST + EPLBD group and in 10 patients in the EST group, but the differences were not statistically significant (EST + EPLBD: 9 % vs. EST: 25 %; *p* = 0.112).

**Conclusions:**

Our results suggest that EST + EPLBD is an effective therapy for patients with difficult-to-treat multiple or large common bile duct stones, because it requires fewer sessions and shorter operative times than EST alone.

## Background

Endoscopic sphincterotomy (EST) is widely recognized as a standard endoscopic treatment for common bile duct stones. However, stone removal with EST alone is often difficult in patients with large or multiple stones, damaged common bile ducts, or tortuous distal bile ducts [1–4]. In 1982, endoscopic mechanical lithotripsy (EML) using a mechanical lithotripter was proposed [5] and proved useful as a concomitant treatment in these difficult-to-treat patients. There is, however, concern that recurrence rates of common bile duct stones increase with the use of ML [6, 7]. In 2003, Ersoz et al. [8] reported the use of EST plus endoscopic papillary large-balloon dilation (EPLBD), and the usefulness of this innovation in patients with difficult-to-remove stones has gradually become evident. This combination is a promising new endoscopic technique for the treatment of common bile duct stones, with efficacy similar to that of EST. However, there is no consensus yet on the usefulness of EST + EPLBD compared with that of EST alone or with concurrent EML. Concrete evidence based on accumulated research findings is needed. This study compares the use of EST with EST + EPLBD and evaluates adverse events in patients with large (≥15 mm) or multiple (≥3) common bile duct stones.

## Methods

### Patients

This study included 70 patients (37 men, 33 women) who had either a single common bile duct stone ≥15 mm or more than three stones. All patients underwent endoscopic treatment between April 2011 and October 2013 at the Department of Gastroenterology, Dokkyo Medical University. We examined the final successful stone removal rate, number of stone removal sessions, successful stone removal rate in the first session, procedure time, status of concomitant ML, and complications.

This study protocol was approved by the ethics committee of Dokkyo Medical University. All patients gave written informed consent before the procedure.

### EST and EST + EPLBD

EST + EPLBD was introduced in our hospital in September 2012. Prior to its introduction, EST was the first-line therapy for multiple or large common bile duct stones. Since its introduction, EST + EPLBD has been used as the first-line therapy in patients, except those who are under 60 years of age and those in whom the distal bile duct cannot be sufficiently dilated.

### Endoscopic technique

Prior to endoscopic treatment, patients were sedated with pentazocine (15 mg) and buprenorphine hydrochloride (3–6 mg). At the time of endoscopic retrograde cholangiopancreatography (ERCP), a protease inhibitor and an antimicrobial agent were administered to prevent pancreatitis and infection [9, 10].

**Fig. 1:**
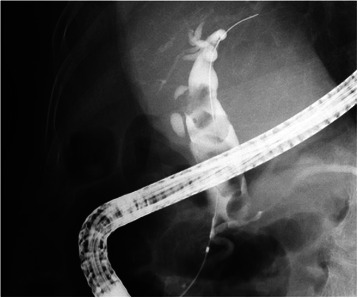
Cholangiogram showing multiple large stones. After the common bile duct was selectively imaged using endoscopic retrograde cholangiography, the sizes and number of stones were confirmed, and the diameter of the distal bile duct was measured simultaneously

**Fig. 2:**
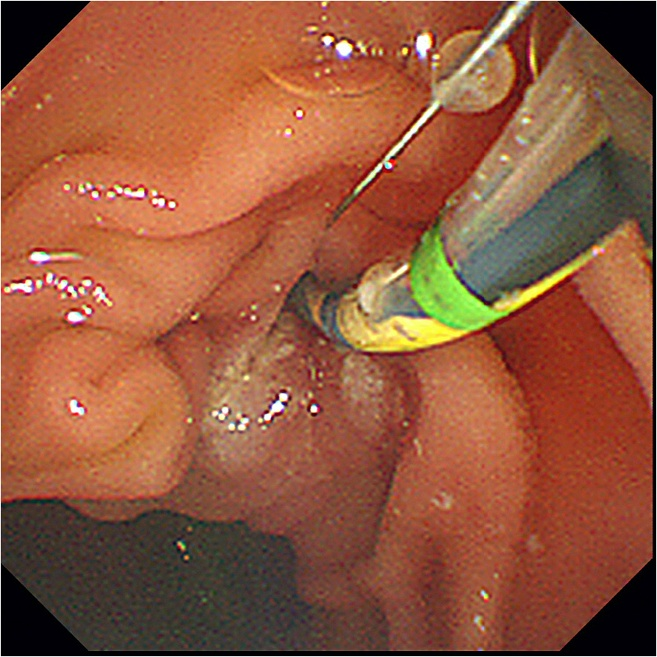
EST before EPLBD. EST was performed before EPLBD in patients who did not receive papillary treatment before balloon dilation

After confirming that the patients were adequately sedated, ERCP was performed using a side-viewing endoscope (JF-260 V, Olympus Medical Systems, Co. Ltd, Tokyo, Japan). At the time of ERCP, an ERCP catheter (MTW Endoscopy, Goldsbergstraße, Germany) was used for contrast-enhanced catheterization and a jag wire (Boston Scientific Japan, Tokyo, Japan) served as the guide wire. After the common bile duct was selectively imaged, the sizes and number of stones were confirmed and the diameter of the distal bile duct was measured simultaneously (Fig. 1). The diameter of the EPLBD balloon (CRE, Boston Scientific Japan, Tokyo, Japan) was selected to correspond to the diameter of the distal bile duct. In all patients, EST was performed before EPLBD (Fig. 2).

**Fig. 3:**
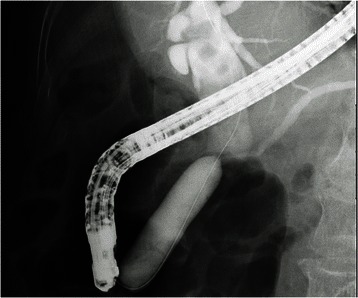
Biliary sphincter dilation with an EPLBD balloon until waist disappearance. After EPLBD balloon insertion in the papilla, the balloon was gradually pressurized until waist disappearance using a special device, and balloon dilation was maintained for 15 s thereafter

**Fig. 4:**
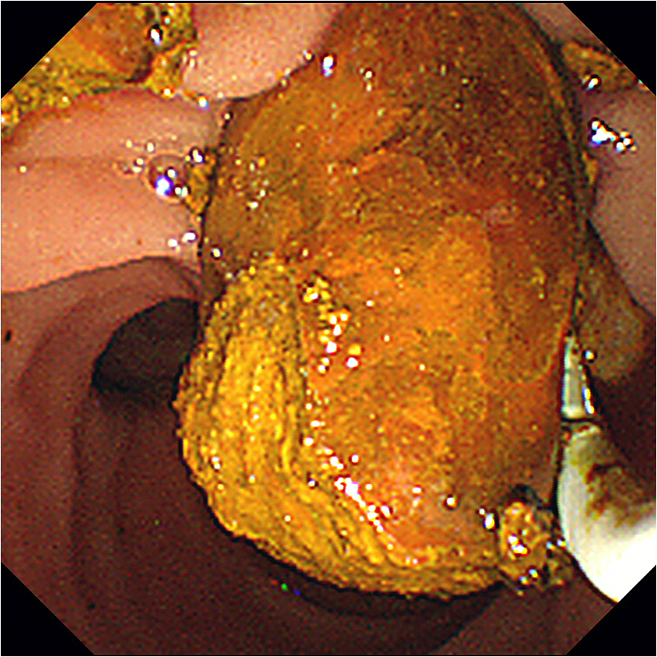
Common bile duct stone removal with a retrieval balloon. The stones were removed after being crushed using a mechanical lithotripter

After EPLBD balloon insertion into the papilla, the balloon was gradually pressurized until waist disappearance using a special device, and balloon dilation was maintained for 15 s thereafter (Fig. 3). The balloon was removed, and the stones were then extracted using a retrieval balloon catheter (Extractor™ Pro RX, Boston Scientific Japan, Tokyo, Japan) or a basket catheter (FG-V425PR1, Olympus Medical Systems, Co. Ltd, Tokyo, Japan). In patients with difficult-to-extract stones, the stones were removed after being crushed using ML (BML-V437QR-30, Olympus Medical Systems, Co. Ltd, Tokyo, Japan) (Fig. 4). To confirm the presence of any remaining stones, the patients underwent contrast-enhanced imaging after occlusion with a retrieval balloon catheter. If the stones could not be removed completely in the first session, another session with plastic stent insertion was performed later.

### Evaluation of stone removal

Stone removal was considered successful when no remaining radiolucent stones were visible on contrast-enhanced imaging after occlusion with a retrieval balloon.

### Procedure time

The operative time was defined as the amount of time required from frontal imaging of Vater’s papilla to the end of the stone removal procedure.

### Evaluation of complications

Post-ERCP pancreatitis, bleeding, and perforation were evaluated. Pancreatitis was diagnosed according to Cotton’s criteria for post-ERCP pancreatitis [11]. Bleeding was defined as a hemoglobin level decrease of at least 2 g/dL from baseline within 24 h after the procedure or bleeding significant enough to require hemostasis. The presence or absence of perforation was evaluated using endoscopic images as well as postoperative survey radiography and computed tomography.

### Statistical analysis

Each variable was statistically analyzed using the chi-square test, Fisher’s exact test, or Student’s *t*-test. A value of *p* < 0.05 was considered statistically significant.

## Results

Table 1 shows the characteristics of the 70 patients in the study, all of whom received endoscopic treatment for a single stone ≥15 mm (largest diameter) or for multiple stones (more than three). Age, sex, mean number of stones, mean diameter of stones, mean diameter of the bile duct, presence or absence of periampullary diverticula, and history of previous cholecystectomy were assessed. No statistically significant differences were observed between the two groups for any of these factors. Eighteen patients (52.9 %) in the EST + EPLBD group and 19 patients (52.8 %) in the EST group had more than three stones. The mean number and mean maximum diameter of stones were, respectively, 5.5 ± 2.6 and 16.6 ± 3.4 mm in the EST + EPLBD group and 4.5 ± 1.7 stones and 18.4 ± 5.6 mm in the EST group.

**Table 1:** Characteristics of patients

	EST + EPLBD (*n* = 34)	EST (*n* = 36)	*P* value
Gender (M/F)	17/17	20/16	0.821
Age (years, mean ± SD)	78.50 ± 1.85	74.19 ± 1.97	0.117
Size of stone (mm, mean ± SD)	18.29 ± 1.02	18.206 ± 0.75	0.944
No of stones (*n*, mean ± SD)	3.50 ± 0.49	2.97 ± 0.34	0.376
Diameter of bile duct (mm, mean ± SD)	15.42 ± 0.49	16.59 ± 0.75	0.198
Periampullary diverticulum (%)	70.59	61.11	0.560
Previous cholecystectomy (%)	8.82	25	0.072
Comorbidities			
Hypertension (*n*)	26	18	0.040
Heart disease (*n*)	8	7	0.901
Cranial nerve disease (*n*)	8	9	0.999
Diabetes mellitus (*n*)	9	7	0.678

Complete stone removal was achieved in all 34 patients in the EST + EPLBD group (100 % stone removal rate) but in only 32/36 patients in the EST group (88 % stone removal rate). This difference was not statistically significant (*p* = 0.115). The EST + EPLBD group showed a higher rate of complete stone removal in the first session (EST + EPLBD: 88.2 % vs. EST: 55.6 %; *p* = 0.003) and a lower mean number of sessions required for complete stone removal (EST + EPLBD: 1.12 sessions vs. EST: 1.47; *p* = 0.002). In addition, fewer patients underwent concomitant ML in the EST + EPLBD group (EST + EPLBD: 50.0 % vs. EST: 94.4 %; *p* < 0.001). The mean procedure time was 66.6 min in the EST group and 42.3 min in the EST + EPLBD group, which indicated a significantly shorter procedure time in the latter (*p* = 0.011). No significant difference was observed in the mean length of hospital stay between the two groups (Table 2). Because the majority of patients underwent treatment for acute suppurative cholangitis and stone removal during a single hospital stay, the length of hospital stay was long. Patients who were admitted at the onset of acute obstructive suppurative cholangitis accounted for 73.5 % of the EST + EPLBD group and 69.4 % of the EST group.

**Table 2:** Comparison between EST + EPLBD and EST

	EST + EPLBD (*n* = 34)	EST (*n* = 36)	*P* value
Complete removal of stone (%)	100	88.9	0.115
No of session	1.1	1.5	0.002
Complete stone removal in 1st session (%)	88.2	55.6	0.003
Use of Mechanical lithotripsy (%)	50	94.4	<0.001
Procedure time (min)	42.3	66.6	0.010
Hospitalization (day)	17.7	20.2	0.160

The cause of stone removal failure in all four unsuccessful EST group patients was the presence of large stacked stones, which resulted in insufficient ML dilation making it difficult to crush the stones. A 7-Fr plastic stent was placed in all four patients and biliary drainage was performed. One patient received surgical treatment. The other three were considered poor candidates for general anesthesia because of advanced age or underlying diseases. These three patients continue to be monitored with regular replacement of their plastic stents.

Table 3 shows complications in all patients. No significant differences were observed in the incidence of bleeding, postoperative pancreatitis, or perforation between the two groups. All patients with pancreatitis had mild cases with the exception of one patient who had moderate pancreatitis, as classified by Cotton’s criteria. All cases of pancreatitis resolved with medical treatment. Bleeding was successfully treated with balloon catheter compression or argon plasma coagulation (APC), and none of the patients required angiography or surgery.

**Table 3:** Complications

	EST + EPLBD (*n* = 34)	EST (*n* = 36)	*P* value
Pancreatitis (%)	5.9	22.2	0.085
Hemorrhage (%)	2.9	5.6	0.999
Perforation (%)	0	0	0.999
Total (%)	8.8	25	0.112

## Discussion

Since the study by Kawai et al. in 1974 [12], EST has been widely accepted as the standard endoscopic treatment for common bile duct stones. However, treatment of common bile duct stones with EST alone is often difficult in patients with large, multiple, or barrel-shaped stones or distal bile duct strictures [1–4, 13, 14].

Several studies have compared the usefulness of EST and EST + EPLBD [13–18]. Some showed no significant difference in treatment results, whereas others reported that EST + EPLBD reduced the operative time, increased the rate of successful stone removal, and reduced the rate of ML use. In 2012, Feng et al. [19] performed a meta-analysis comparing EST and EPLBD. According to their findings, the successful stone removal rate in patients with common bile duct stones treated with EPLBD was 97.35 %, 87.87 % were successful in the first session, indicating favorable results. However, the differences between EPLBD and EST were not statistically significant. In addition, in patients with large stones, EML use did not differ significantly between the two groups, and the occurrence rate of bleeding, an early accidental event, was significantly lower in the EPLBD group. No consensus has been reached based on previous studies.

In our study, there was no difference in stone removal rates, which is consistent with the earlier reports [13–18]. In contrast, our study showed that the complete stone removal rate in the first session was significantly higher in the EST + EPLBD group than in the EST group. In addition, EST + EPLBD use required significantly fewer sessions to achieve complete stone removal. Although these results were the same as those of Kim et al. [16], no differences were noted between EST + EPLBD and EST in the other studies [15, 18]. Kim et al. [16] attributed failure of complete stone removal in the first session to the presence of large and/or multiple stones. Differences in previous study results are likely attributable to the differences in the sizes and numbers of stones in the patients examined. Without the concurrent use of ML, it is often difficult to remove large stones using conventional EST alone. Moreover, a larger incision of papillary muscles is required, which increases the risk of serious complications such as bleeding and duodenal wall perforation [13, 14, 20–22]. Therefore, large stones can usually be more safely and securely removed using conventional EST with ML for stone fragmentation [14, 23]. However, incomplete removal of stone fragments is a risk factor for recurrent stones [6, 7] and stone removal using ML often fails in patients with large stones [16, 24, 25]. In our study, the rate of ML use was significantly lower with EST + EPLBD than with EST. Although similar results have been reported by other investigators [8, 13, 16, 26], there was no difference in the rate of ML use between the EST and EST + EPLBD groups in a previous study [18]. In previous studies comparing EST and EST + EPLBD, the rates of ML use during EST ranged from 9 to 33 %, showing a marked difference from the 94 % rate of ML use in the present study. This may be because the mean diameter of the common bile duct stones was larger in our study than that in previous studies. Our results suggest that EST + EPLBD decreases the frequency of ML use for large or multiple stones, decreasing the number of remnant stone fragments that cannot be confirmed with endoscopic retrograde cholangiography. However, in patients with large stones and a tapered lower common bile duct, stone removal is often difficult with concurrent EST + EPLBD without ML use. Therefore, ML use is determined by bile duct form and stone diameter.

Operative time was also shorter with EST + EPLBD than with EST alone. This result is valid considering the rate of complete stone removal in the first session and the rate of ML use. Taken together, all of our results suggest that EST + EPLBD can achieve greater dilation of the bile duct opening than conventional EST alone, thus facilitating stone removal.

Complication rates did not differ significantly between the EST and EST + EPLBD groups. Kim et al. reported that the rate of accidental events in EST + EPLBD patients was 8.3 % (0–17.0 %) and the incidence of pancreatitis was 2.4 % (0–13.2 %), consisting mostly of mild-to-moderate pancreatitis [27]. Our results showed no occurrences of serious pancreatitis in the EST + EPLBD patients, and the incidence of all complications was 8.8 %, which is comparable to the rates reported in previous studies. No significant differences in the occurrence of pancreatitis were observed between the EST and EST + EPLBD groups. The similarity in pancreatitis rates in the EST + EPLBD and EST groups is attributable to the reduced effects of the separation of the pancreatic duct from the biliary orifice by EST before balloon dilation, indicating that additional EST before EPLBD may decrease the incidence of pancreatitis [13, 16, 18, 26]. Bleeding occurred in one patient in the EST + EPLBD group and in two patients in the EST group. Hemostasis was achievable in both groups by APC or compression with a retrieval balloon, and there were no significant differences between the two groups. However, Ersoz et al. [8] reported that the bleeding rate in patients receiving EST + EPLBD was 9 %, with the risk of bleeding being particularly high in those with a tapered distal bile duct. In such a case, balloon dilation should be performed only after careful consideration. We advocate morphological evaluation and diameter measurement of the distal bile duct because these data are important for performing EST + EPLBD. This recommendation is also intended to prevent perforation, which did not occur in our study.

Treatment using EST reduces the function of Oddi’s sphincter and may cause long-term problems such as recurrent bile duct stones and repeated retrograde cholangitis [28]. Since EST + EPLBD can theoretically achieve greater dilation of the bile duct opening than EST, the decrease in postoperative Oddi’s sphincter function is considered to be equivalent to or greater than that observed with EST. Therefore, further study of long-term outcomes is required.

## Conclusion

The results of the present study show that EST + EPLBD allowed complete stone removal in fewer sessions and in a shorter time frame as compared to EST, without increasing the number of accidental events in the treatment of large (≥15 mm) or multiple (≥ three) stones. The final successful treatment rates did not differ between the EST + EPLBD and EST groups, suggesting that the indications for EST + EPLBD should be determined with care. Future studies with larger sample sizes for more detailed examination, including assessment of long-term outcomes, are necessary.

## Abbreviations

EST: Endoscopic sphincterotomy
EPLBD: Endoscopic papillary large-balloon dilation
EML: Endoscopic mechanical lithotripsy
ML: Mechanical lithotripsy
ERCP: Endoscopic retrograde cholangiopancreatography
APC: Argon plasma coagulation
